# The experience of adults with multimorbidity: a qualitative study

**DOI:** 10.15256/joc.2014.4.31

**Published:** 2014-05-28

**Authors:** Cynthia Duguay, Frances Gallagher, Martin Fortin

**Affiliations:** ^1^Faculty of Medicine and Health Sciences, Université de Sherbrooke, Sherbrooke, Quebec, Canada; ^2^Nursing School, Université de Sherbrooke, Sherbrooke, Quebec, Canada; ^3^Department of Family Medicine and Emergency Medicine, Université de Sherbrooke, Sherbrooke, Quebec, Canada, and Centre de santé et de services sociaux de Chicoutimi, Chicoutimi, Quebec, Canada

**Keywords:** qualitative study, adult, multimorbidity, experiences, primary healthcare

## Abstract

**Background:**

Findings from several countries indicate that the prevalence of multimorbidity is very high among clients of primary healthcare. A deeper understanding of patients’ experiences from their own perspective can greatly enrich any intervention to help them live as well as possible with multimorbidity.

**Objective:**

To describe the fundamental structure of adults’ experience with multimorbidity.

**Design:**

A phenomenological study was undertaken to describe the experiences of 11 adults with multimorbidity. These adults participated in two semi-structured interviews, the content of which was rigorously analyzed.

**Results:**

At the core of the study participants’ multimorbidity experience are the impression of aging prematurely, difficulties with self-care management, and issues with access to the healthcare system, which contribute to the problem’s complexity. Despite these issues, participants with multimorbidity report attempting to take control of their situation and adjusting to daily living.

**Conclusions:**

The description of this experience, through the systemic vision of participants, provides a better understanding of the realities experienced by people with multimorbidity.

Journal of Comorbidity 2014;4:11–21

## Introduction

The manner in which people conceptualize their health status, in combination with the emotional aspects of the lived experience, directly influences their experience, including their adherence to treatment plans and medical recommendations [1]. People living with several chronic conditions (multimorbidity) require healthcare interventions that represent a considerable challenge for both the individuals themselves and for primary care workers. The high prevalence of multimorbidity and the relationship with age have been described in several publications and have raised much attention [2–6]. Multimorbidity is associated with lower quality of life [7, 8] and higher psychological distress [9]. The management of multiple self-care interventions represents a real challenge for these patients [7, 10, 11]. Only a few studies have examined the experience associated with multimorbidity, and these were primarily in older people [11–17].

The results of these studies indicate that the many challenges associated with multiple chronic diseases represent more than just the sum of each individual’s diseases [11]. People with multimorbidity perceive their state of health as a series of medical and emotional crises that inevitably lead to physical loss and limitations, and can also create difficult social relationships [16].

To date, the literature does not provide sufficient evidence for us to determine whether the experiences of adults with multimorbidity are similar to those of the older population, despite multimorbidity also being common in younger adults [2], whose social and family situations differ from those of their elders. It is important to continue developing our understanding of patients’ experience of multimorbidity, so we can implement interventions adapted to our patients’ needs and goals. The purpose of this phenomenological study is to describe the fundamental structure of adults’ experience with multimorbidity.

## Methods

### Design of the study

Descriptive or transcendental phenomenology [18] provides a framework within which we can describe the meaning of the multimorbidity experience among adult primary care clients. Using this approach, researchers make sure to set aside (bracketing) their own knowledge and experience of multimorbidity, and better understand the experience from the participants’ perspective [19]. An interdisciplinary triangulation of researchers (one physician, two nurses) provides a broader disciplinary perspective.

### Participants

The study took place in Canada, in a French-speaking region of Quebec, with a sample of 11 adults receiving primary care follow-up. To recruit participants, we obtained the collaboration of seven family physicians working in three family medicine clinics. We received the name and phone number of 12 adults who showed an interest in the study; only one person did not match our criteria. The participants, men or women, had to be between 18 and 69 years of age and have at least four chronic diseases, to reflect the multimorbidity experience among adults. At the time of the study, women could not be pregnant and all participants had to have a life expectancy of more than 1 year (including those with a terminal illness), to avoid confusion between these experiences and that of multimorbidity. People with moderate-to-severe cognitive impairment, decompensated psychiatric illness, or a hearing problem and no hearing aid were also excluded. The research received approval from the ethics committees of the clinics where the research took place and from the Faculty of Medicine and Health Sciences, Université de Sherbrooke, where the first author was studying. Following this approval, the first author phoned the adults referred by a family physician to explain the study in detail and answer their questions. They gave verbal consent on the basis of the information presented. An appointment was then made for an interview with those who agreed to participate in the study. At the first face-to-face meeting, the informed consent form was read and written consent was given for the interview and for the follow-up interview. However, when contacting participants by phone before the second interview, the first author verified that they still agreed to participate in the interview. This assured confidentiality and allowed voluntary withdrawal from the study without the necessity of providing an explanation or of any prejudice from their family physician.

### Procedures

We collected the data in three stages: (1) a semi-structured interview with participants, which included a sociodemographic questionnaire; (2) a second semi-structured interview a few weeks later; and (3) the collection of additional information from the family physician about the chronic diseases of participants. During the first participant interview, we attempted to get a sense of the multimorbidity experience. The interview guide included open-ended questions, such as: What does it mean to you to have several chronic diseases? What images come to mind when you think about your experience with chronic diseases? What feelings do you have about living with several diseases at the same time? We also used probes when necessary, for example: Can you explain your thinking to me in more detail? During the second semi-structured interview, which took place 4–6 weeks later, we validated, among participants, our understanding of the content of the first interview. We also asked questions to further explore the participant’s point of view, such as: At our first meeting, you mentioned that you felt your diseases limit you. I would like to better understand what kinds of limitations arise from your chronic diseases and what these limitations represent for you. The first interview lasted approximately 60 min, and the second, 40 min. We recorded the interviews in a digital audio format, and took notes throughout the data-collection process to document nonverbal communication and context. We provided those study participants who preferred not to be interviewed in their own home with minimal financial compensation for transportation costs. In the third stage of data collection, we contacted the family physician of each participant to ask for a list of the participant’s chronic diseases, which was subsequently sent to us by e-mail or fax.

### Analysis

We analyzed the content of the interviews according to the inductive method proposed by Colaizzi [18], to determine the meaning and structure of the essence of the multimorbidity experience. The purpose of phenomenological research is “to capture as closely as possible the way in which the phenomenon is experienced” [20]. The phenomenology is used to look for the meanings that constitute the phenomenon in the participant’s lifeworld. The idea is to study how individuals live; that is, how they behave and experience situations [21]. The first author (C.D.), a graduate student, conducted each stage of the analysis first and then reviewed and discussed it with her co-authors (M.F. and F.G.). To minimize the impact of personal bias on her analysis, she (C.D.) wrote a reflective journal about her values, beliefs, and experiences relating to multimorbidity, so that she could more effectively set them aside and identify the core of the meaning of adult multimorbidity. Throughout the research process, all authors discussed and reiterated the importance of disassociating their own perceptions from the participants’ experiences. We transcribed the content of each interview verbatim.

The first stage of the analysis consisted of listening to the interview recording while simultaneously reading the transcript and observational notes of any nonverbal communication, to understand the overall meaning of the participants’ comments. In the second stage, we identified significant excerpts describing the multimorbidity experience according to the participants’ point of view. We later repeated these first two stages with each participant’s second interview. Subsequently, we transformed the significant excerpts into more general significant statements, which helped eliminate any redundancy. For example, in the first stage of the analysis process, some participants talked about their fears of pain, of possible serious complications, and of “being a burden, especially in a few years”. These significant statements were transformed into the following more general statement: Uncertainty and anticipation related to the fluctuations of one’s health status. Supplementary Table 1 presents a list of these significant statements. In the third stage, the purpose of which was to discover the meaning of the significant statements [18], we formulated meanings of significant statements (Supplementary Table 2). In the fourth stage, we organized these statements of meaning into themes and sub-themes describing the multimorbidity experience, promoting the emergence of themes common to all participants. To ensure that the themes accurately captured the meaning of the multimorbidity experience that participants described, we reviewed the original excerpts within the framework of phenomenological reduction. We were careful not to exclude too quickly any data that might have seemed contradictory or not interconnected. In the fifth stage of analysis, we developed a comprehensive description of the multimorbidity experience, based on the themes and sub-themes that we identified in the preliminary stages. In the sixth and final stage of analysis, we transformed this description into an unequivocal statement of the essential structure of the experience. At this stage of analysis, a reduction of findings was done in which overestimated descriptions were eradicated from the overall structure. Some amendments were applied to generate clear relationships between clusters of themes and their extracted themes, which involved eliminating some ambiguous structures that weaken the whole description. We began to find data redundancy by the seventh participant. At this point, no new data were forthcoming.

## Results

The sociodemographic characteristics of the participants are presented in Table 1. The mean age was 58.1 years (range 37–66 years) and the proportion of men was higher (64%). The average number of chronic conditions was 7 (range 5–11). The prevalence of the most commonly reported conditions was: hypertension (91%), hyperlipidemia (91%), diabetes (73%), coronary artery disease (64%), obesity (55%), arthritis (45%), and chronic obstructive pulmonary disorder (27%). There was no specific combination and the small number of patients precludes the identification of any combination of chronic conditions for the sake of confidentiality.

### Multimorbidity experience

The fundamental structure of the experience of living with several chronic medical conditions involves five main themes: (1) the impression of premature aging, even in young adults; (2) the unique nature of the experience of multimorbidity; (3) complex management of multimorbidity; (4) psychological burden; and (5) ownership of multimorbidity. Table 2 presents these themes and the sub-themes they comprise.

(1) Impression of premature aging

A feeling of premature aging is one of the primary characteristics of the experience of living with several chronic diseases. Regardless of the participant’s age, in their eyes, chronic diseases and the numerous associated physical and social limitations evoke old age.

#### Chronic diseases symbolizing old age

Participants had the impression of aging more rapidly than healthy people of their own age, and of feeling older than their true biological age. Participants’ statements to this effect were unambiguous, regardless of whether they had five or even 11 chronic diseases, for example: “The image that comes to mind when thinking about chronic disease is old age” (male, early sixties, five chronic conditions). Participants associated the accumulation of chronic diseases with a phenomenon that occurs in very old people and not in adults of their own age.

#### Lived experience linked to the loss of physical and social abilities

People with multimorbidity face many physical and social losses, and they often consider their diseases to be a “general handicap.” They relate that they perceive their body as something fragile and limiting, as one participant explained so eloquently: “I’m like a butterfly, because my health is ephemeral, and just like a butterfly, I have to land and rest, because I’m less able to get things done” (female, early sixties, six chronic conditions). This feeling of fragility and slowing down of physical functioning occurs in those who otherwise consider themselves to be young. The physical limitations their health imposes — such as respiratory problems, joint pain, or lack of muscular endurance — affect their ability to perform many daily activities and even to hold a job. A father with obesity, chronic lower back pain, and osteoarthritis (among other diseases), which cause problems with mobility, mentioned that: “When it comes to my work, it’s really hard, I’m limited physically in what I do compared with what I was doing before, not easy at my age” (male, late fifties, five chronic conditions).

Physical limitations also affect participants’ social lives, because they are constantly worrying about disease management (medication, treatment, restriction), even while participating in social activities, and could feel excluded. “My cousin has a cabin in the woods 2 hr away and doesn’t dare invite me anymore because of my health” (female, in her thirties, six chronic conditions). In general, participants in this sample preferred to do things alone rather than to deal with the pressure of spending time with others. Finally, some participants said that multimorbidity also affects their sex life, another dimension of social life: “I find it difficult not being able to do the same activities as before, both in terms of going out and having sex” (male, early sixties, five chronic conditions).

(2) Unique nature of the experience of multimorbidity

According to the participants, living with several chronic diseases is a particular situation that is more complex than having a single chronic disease. The unique nature of the experience of multimorbidity translates into many disadvantages, such as feeling caught in the sequence of several concomitant diseases, living with multiple interactions (disease–disease; treatment–disease; treatment–treatment), and the complex management of multimorbidity.

#### Caught in the sequence of several concomitant diseases

All participants had internalized the idea that the experience of multimorbidity is not confined to the occurrence of a single disease; rather, they felt like a new disease was lying in wait for them at every turn. For example: “What I find annoying is that it doesn’t stop, there’s always something new that appears” (male, early sixties, eight chronic conditions). They were caught in a vicious cycle of diseases that accumulate one after the other, as this excerpt illustrates: “Before my back injury, I was in good shape. Before my depression, I was in good shape. Before my heart attack, I was also in good shape. Today, all that has changed. To try to move quickly, to recover what I was doing before, it’s more difficult, it’s more complicated” (male, early sixties, six chronic conditions).

#### Multiple interactions: disease–disease; treatment–disease; treatment–treatment

The presence of several health problems, in a system as complex as the human body, results in a variety of interactions. All participants internalized these interactions as another dimension of the multimorbidity experience, regardless of current health conditions. Relationships between the chronic conditions themselves come in various forms, with both psychological and physical components: “I had back pain and wasn’t able to recover physically, because I didn’t sleep much at night [I was stressed]. In addition to not recovering, I was stressed by that. I was feeling blue more and more, which certainly didn’t help my morale or the [occurrence of] depression. I’m sure that also contributed to my heart problem” (female, mid-fifties, nine chronic conditions).

The above comment illustrates the fact that the multimorbidity experience is an overarching experience that is not restricted to the sum of the individual diseases. In addition to interactions between health problems, this experience was also due to the involvement of treatment-related interactions, which can be very problematic in daily life. One participant touched on the relationship between the treatments prescribed for arthritis, cardiovascular problems, and chronic pain, and the side effects of these treatments: “Everything interconnects; there is no drug that doesn’t have a small side effect that affects something else. Anti-inflammatory and antibiotic drugs get into my stomach and I have ulcers. So I endure the pain. [Drug A] and [Drug B] raise my sugar level. Let’s say I want to buy myself a treadmill to exercise so that I can improve my strength. I can’t do it; I’ll have too much pain in my knees. I’ll still have some pain everywhere” (male, early sixties, five chronic conditions).

The multiple medications (polypharmacy) required for the treatment of multiple chronic diseases cause other problems for most participants, adding to the complexity of the situation. For example, some drugs have side effects that require treatment with other drugs, which contributes to increasing polypharmacy. This situation occurs frequently contributing to the sense of the experience related by participants: “I took [Drug C] and another drug because I had stomach pain from my 12 pills” (male, mid-sixties, eleven chronic conditions). Discomfort, such as fatigue or pain associated with a chronic disease, can also represent a barrier to the implementation of the self-care required for another disease. Thus, participants noted they felt they were caught in a cycle of interactions: “It’s a wheel that turns; if I have edema, I’ll run less, I’ll be less inclined to want to do physical activity, which doesn’t help my other health problems” (male, early fifties, six chronic conditions).

#### Diseases experienced differently depending on the presence or absence of pain

Not all diseases have the same significance in the overall multimorbidity experience. Those that involve pain are more distressing than those whose symptoms are more difficult to detect: “My joint pain is more distressing [compared with hypercholesterolemia and obesity], because I feel it every day walking, at work, in all activities, whereas the cholesterol doesn’t hurt. I take a pill. I don’t worry as much” (female, mid-sixties, ten chronic conditions). In the absence of pain or noticeable symptoms, diseases such as diabetes, hypertension, and dyslipidemia can go unnoticed in everyday life. Even with nine diseases, one participant said: “Day-to-day my life is normal. The only time I think about having diseases is when I’ve just met with the doctors” (female, mid-fifties, nine chronic conditions).

(3) Complex management of multimorbidity

Self-management of drugs, treatments, and medical appointments can be perceived as being difficult, even with a single chronic disease. The multimorbidity experience involves the complex management of several chronic health problems, which have a major impact on everyday life. Complicated access to the healthcare system intensifies the experience associated with multimorbidity.

#### Daily burden of multimorbidity

Multimorbidity, in all its complexity, has multiple implications in various spheres of everyday life. Self-management of many chronic diseases is disruptive and represents an additional daily burden because of treatments, required self-care, medical follow-ups, and management of polypharmacy: “I had to reorganize my way of thinking, my way of acting. Reorganize my activities to be able to do as many as possible. Drop some of them to compensate. Having to compensate for not being able to do everything brings stress, the stress of managing my time and handling my activities to be sufficiently in good shape to continue what I was doing before” (male, early sixties, five chronic conditions).

Multimorbidity, and the diseases and treatments that characterize it, brings its share of difficulties and requirements for the study participants. They all felt the need to adopt a very strict discipline to carry out the self-care their condition requires. They have had to change their usual ways of doing things, which can be an additional source of stress. Among the components of the treatment plan, one of the major challenges was weight loss and all the self-care it requires: “It’s not easy to put yourself on a diet, and it’s also not easy to follow it. Is this good for me? It’s not good. My wife went again to take classes to learn how to read labels” (male, early fifties, six chronic conditions). “To improve my condition, I also had to quit smoking and lose weight. I said I’ll go on a diet, but I won’t stop smoking. Well forget that. Now I realize that quitting smoking would be easy compared with following a diet” (female, late thirties, six chronic conditions).

Adding to the daily burden of multimorbidity, the management and obligation to take multiple drugs at the same time, several times a day, is related as an everyday reality: “Many chronic diseases require treatment with many drugs” (female, early sixties, six chronic conditions). One female participant even remarked, laughing, that she does her grocery shopping at the pharmacy and then purchases a few provisions at the grocery store.

The management of multimorbidity also includes self-care related to medical follow-up that requires frequent medical visits, sometimes with many specialists, which participants experience as an additional load. Traveling to various medical clinics and planning various appointments adds to the complex nature of multimorbidity management and its daily burden: “There are so many appointments associated with my disease, and that bothers me” (male, early fifties, six chronic conditions).

#### Difficulties with access to the healthcare system

Poor access to the healthcare system contributes to complicating the management of multimorbidity. Many health problems require medical follow-up, as well as occasional emergency visits. Participants find that making an appointment with their family physician or with medical specialists is difficult: “That’s what’s most complicated, what’s hardest: Just making an appointment with my doctor” (female, mid-fifties, nine chronic conditions). Obstacles include overloaded telephone lines at medical clinics and the inability to schedule an appointment less than several months in advance.

Place of residence also impacted the multimorbidity experience of some participants, by either facilitating or complicating access to health services: “I live in the suburbs, which means more travel for my appointments and more costs. I’m seriously thinking of moving closer to the hospital” (male, early sixties, five chronic conditions). Problems with access to the healthcare system figured very prominently in the description of participants’ experience. One participant commented: “It’s not the disease that I’m fighting; it’s the healthcare system” (male, early sixties, six chronic conditions).

#### Need for support in treatment follow-up

Living with several chronic diseases can certainly represent a difficult experience, and several participants expressed that they needed support to implement the self-care that their situation required. Family members, especially spouses, often play a leading role in sharing responsibility for some of the care. Participants also reported that they needed to feel encouraged for their efforts. For participants living alone, healthcare professionals can help to meet this need for support: “Physicians, nurses, nutritionist… uh… It helps, especially when you’re alone. It’s the loneliness. You don’t feel like talking, and then you find that when you talk about it, you talk too much. Me, I don’t have a spouse, I’m all alone. I don’t have anyone to support me with my new diet. You know, to help me live with it and encourage me” (female, mid-fifties, nine chronic conditions).

(4) Psychological burden

The multimorbidity experience has a variety of negative emotional impacts, and participants generally live with the ongoing anticipation of possibly having to deal with additional health problems in the future.

#### Negative emotional impacts

Living with several chronic health problems and the associated limitations was perceived as the cause of unpleasant feelings and emotions: “Feelings of anger, disgust, and discouragement when I’ve failed to do what I would like to do, because my diseases prevent me” (female, mid-sixties, ten chronic diseases).

Powerlessness, or the feeling of not having control over the situation, is often part of the multimorbidity experience. Participants felt diminished “physically and morally” because of the loss of autonomy associated with multimorbidity. This feeling was especially strong for participants who had to depend on others to perform the daily tasks of domestic life. Some self-care, such as that associated with polypharmacy, reinforced participants’ negative perceptions of their health status, increasing their sense of worthlessness. One 52-year-old participant said: “I had never taken medication, now I always have the dosette box there, next to me on the table. It’s far from being rewarding, it’s even worse than you think it will be” (male, early fifties, six chronic conditions). Some participants felt a deep sense of despair because their situation was so stressful, and they might even have already considered suicide.

#### Uncertainty and anticipation related to fluctuations in health status

Uncertainty and anticipation of problems occupied an important place in the description of the multimorbidity experience. Indeed, participants said they were living with ongoing uncertainty associated with the evolution and fluctuations of their health condition: “With disease, you can’t predict the degree to which it might be disabling” (male, early sixties, five chronic conditions). One participant used a metaphor to depict his situation: He felt he was sitting on a shaky chair, not knowing whether it would collapse or stay in place. Participants anticipated the occurrence of other problems and feared the possibility of losing their power to act and make decisions. They also anticipated the burden that would fall on their family once they lost the capacity to meet their own needs.

(5) Ownership of multimorbidity

Several themes supported the complexity of the experience associated with multimorbidity, other themes reflected participants’ ability to take “ownership” of their diseases, such as by making day-to-day adjustments to health problems and implementing self-care beneficial to the simultaneous management of several diseases.

#### Day-to-day adjustment to health problems

Adjusting to the constant presence of health problems involves a change in thinking and behavior. “You need to adapt to health problems when performing the activities of day-to-day living” (male, mid-sixties, eleven chronic conditions). Experiencing multimorbidity means living and evolving with the diseases. These adjustments had eventually formed an integral part of everyday life for participants: “Chronic diseases mean adjustment. I have highs and lows, it’s normal, and it’s part of life. You have to continue to adjust” (female, mid-sixties, ten chronic conditions).

Following diets specific to their diseases, modifying day-to-day and social activities according to a treatment schedule, and learning to live with physical limitations were some of the adjustments participants had made. These adjustments were based on a good understanding of their health status and a commitment to take charge of their own care. Learning to live with chronic diseases gave meaning to the multimorbidity experience and encouraged many participants to take ownership of their situation: “When we have problems, there is no point in dwelling on our fate; we have to organize ourselves accordingly and accept it” (male, mid-sixties, six chronic diseases).

#### Benefits of self-care

Although many types of interactions contribute to the complexity of the experience of multimorbidity, some interactions are actually perceived as beneficial. A single self-care intervention can have a positive snowball effect on several diseases. One participant with eight chronic diseases described this sequence of positive effects very well: “Consider losing a great deal of weight; it helps a lot to reduce the effects of sleep apnea. This is often a big issue. If you’re physically in shape, you exercise, your blood sugar stabilizes. Even if you don’t eat properly at every meal; the exercise compensates for it. It’s the same thing with asthma. If you exercise and have a good workout, your breathing will improve and you’ll be able to control it better. That’s it. That’s why I said it’s a wheel that turns. So if you try to improve one health problem, you’re automatically going to improve the others” (male, early sixties, eight chronic conditions).

The ability to take ownership of one’s situation and recognize the multiple benefits of a self-care behavior requires an understanding of the links between diseases. Self-care related to pharmacologic treatment is another source of benefit and represents an essential component of the experience, notably for its reassuring effect: “With medication, I’ve struck gold. It’s reassuring” (male, mid-sixties, eleven chronic conditions). In general, carrying out the self-care actions that their health status requires brings a healthy dimension to participants’ multimorbidity experience. Three of the participants had the impression of being healthy patients because of their medication and health behaviors: “I exercise because I’m sick, and I take drugs because I’m sick, but these three together [exercise, drugs and illness] make me a sick person who’s healthy” (male, early sixties, five chronic conditions).

#### The structure of experience

The culmination of a phenomenological study is the identification of the essential structure of the studied experience. The themes presented in this paper show that within the structure of the multimorbidity experience, there is an impression of aging prematurely because of many physical and social losses. This feeling of premature aging is complex, involving a sequence of several diseases and a multitude of interactions requiring complicated management. Despite the major psychological burden, participants tended to take ownership of their multimorbidity and adjust their daily lives according to their health status and the care and self-care their health condition requires.

## Discussion

### Impression of premature aging

The results presented here extend prior research on multimorbidity by describing the fundamental structure of this experience for an adult population; whereas, to date, researchers have conducted the majority of studies on this subject among the elderly [12, 13, 22]. One of the main findings of this study concerns the impression of premature aging, which was at the heart of the multimorbidity experience, regardless of participants’ age. Participants felt older than their biological age, an impression based on the perceived association between old age and the accumulation of several health problems. Participants had an average of seven chronic diseases. Thus, we see that the presence of several chronic diseases modulated participants’ relationships with their bodies. Findings from other studies complement this result by describing a reverse situation, emphasizing that biological age is not always a good indicator of body perception or function. A 70-year-old man, active and at a healthy weight, can have the cardiovascular fitness of a man 10–15 years younger [23]. In this light, in a study of people between 40 and 74 years of age (mean age 54.3 years), people who perceived themselves as healthy felt younger [24].

A decrease in overall ability naturally accompanies aging [25], therefore it is not surprising to find a feeling of old age in participants with chronic diseases, particularly when these diseases are associated with disabilities. The results of our study are consistent with the experience of people with multiple chronic diseases as reported in the literature. Other authors have documented the physical limitations that prevent the performance of activities and create the need for assistance [11, 13, 17], as well as the numerous types of loss in ability that result in, among other things, loss of employment [15]. In a society that values performance and profit [26], maintaining social roles may be more important to patients than managing symptoms [17].

### Systemic view of the multimorbidity experience

A system is a set of elements in standing relationship according to certain principles that the nature of the various elements determines [27]. Participants described the unique nature of the experience of multimorbidity, complex management of care and self-care, and psychological burden from a systemic perspective. They explained the unique nature of the experience of multimorbidity by talking about the many interactions between treatments and diseases, as well as by emphasizing the sequence of their diseases. Schoenberg and colleagues [15] concluded that multimorbidity is not limited to the sum of the individual diseases, but is a complex phenomenon in itself. Other authors have pointed out various levels of interaction, because every disease can affect other diseases or treatments, and each treatment can interact with other treatments or can even lead to other diseases [28].

Despite having a generally consistent overall perception of the multimorbidity experience, it seems that participants also experienced diseases differently, depending on whether they caused pain or not (for example, arthritis vs. an increase in cholesterol levels). Diseases with pain are more disruptive on a day-to-day basis, as Roberto and colleagues noted in their 2005 study [13]. The literature on multimorbidity has focused little on the dimension of pain in the experience associated with this health condition. Considering the impact of pain on day-to-day activities, it would be appropriate to pursue the subject to better take into account this aspect of multimorbidity.

Participants emphasized the multiple elements that they had to deal with to assume management of their health situation, which they called “complex”. They noted, among other things, the aggravating effects of the drugs or diet required for one of their illnesses on another health condition. In studies on multimorbidity, authors have corroborated the complexity of managing diseases and treatments [12, 15]. Indeed, management of a single chronic disease has been described as “endless work” [29]. Considering the complexities of participants’ situations, it is not surprising that they mentioned that management of their care and self-care frequently required them to reorganize their daily lives.

It is clear that the structure of the multimorbidity experience rests on a systemic perception. This supports the relevance of a comprehensive approach during interventions among people with multimorbidity. However, the guidelines for monitoring people with chronic diseases are distinct for each chronic disease. Instead of focusing on the treatment and care of each specific disease, guidelines should include a holistic approach and interventions that take into account the comprehensive nature of the multimorbidity experience [30].

Similarly, best practices in primary care focus on patient’s involvement in their own care and decisions that concern them [31]. They include tailored interventions that take into account an individual’s attitudes, beliefs, and preferences. This orientation of care requires an exploration of an individual’s interpretation of their disease, their impressions, and their personal circumstances. Patient-centered care focuses on close collaboration with patients. Our results illustrate the specificity of the multimorbidity experience and support the importance of a patient-centered approach, capturing the complexity of the meaning of multimorbidity experience. This type of primary care approach should be the default course of action for people living with multimorbidity [30].

For study participants, management of their condition included issues related to problems accessing the healthcare system. In this study, we illustrate how disturbing and worrisome these accessibility issues can be to patients with multimorbidity. An international study involving adults 18 years of age and older in 11 different countries showed that people with multimorbidity had more difficulty with access to and coordination of care [4], a finding that supports the results of our study. These findings about the barriers to accessibility of care highlight the need to review the structure of primary care services and service corridors for people coping with several chronic diseases.

Psychological burden was one aspect that characterized the systemic perception of the multimorbidity experience among participants, whether manifesting as anger, feelings of worthlessness, or feelings of powerlessness in coping with physical limitations and treatments. These unpleasant feelings are consistent with what other authors have observed in people coping with several chronic diseases [13, 17]. Some authors have found that patients experience a decrease in quality of life and an increase in psychological distress [7, 9], underlining the importance of intervening, using a patient-centered approach, and not neglecting this aspect of the multimorbidity experience.

### Ownership of multimorbidity

It is clear that participants had to make day-to-day adjustments according to their health status to take ownership of their diseases. Many participants chose to maintain a positive attitude and focus on the diseases over which they had power, by employing strategies conducive to taking ownership of their health status. A positive attitude has a very beneficial effect on one’s ability to manage the activities of daily living [32]. In addition, taking care of oneself in compliance with the recommended treatment plan generates a sense of control over one’s health situation [12]. Commensurate with other research findings, participants tried to take ownership of their diseases, to see their state as “normal” [17], and to maintain some independence [13].

The multimorbidity experience also included a series of beneficial effects associated with daily self-care. For one participant, following the dietary recommendations for diabetes led to weight loss, which reduced the effects of apnea and asthma, resulting in a sense of physical and psychological well-being. Some participants considered themselves to be healthy patients, because of their medications and health behaviors. Paterson’s [33] shifting perspectives model of chronic illness holds that the shifting perspective process of chronic disease allows people to give meaning to their experience; one can perceive chronic disease as an opportunity to make a significant change in one’s life and in one’s relationships with others. People try to establish harmony between their own identity and the identity associated with the disease, and they typically have a very optimistic opinion of their situation [33], as we have seen in our study participants.

The strengths of this study include our use of two separate, in-depth interviews with each participant. An interdisciplinary triangulation of researchers (one physician, two nurses) provided a broader disciplinary perspective and contributed to the rigor of the research process. We rigorously adhered to the principles of bracketing and phenomenological reduction [19], ensuring better reliability of the results. The sample size of 11 participants could be considered a limitation, but in phenomenology, a sample size of between 5 and 25 participants is acceptable [34]; the participants, all French-speaking, were recruited in only one region of Quebec. This may limit the applicability of the results to other populations.

## Conclusions

We illustrate that people with multimorbidity live an experience that is associated with the impression of premature aging, which they describe according to a systemic perception. Knowledge of these results should inspire healthcare system managers and clinicians to advocate a systemic approach to care and service provision to adequately meet these patients’ lived experience of multimorbidity. Inquiring about what multimorbidity means to a patient, might be an efficient method for nurses and physicians in primary care settings to assess a patient’s illness representation and coping processes, and enhance nurse–patient and physician–patient relationships. Future studies should continue to explore these experiences and the development of interventions tailored to the realities of people with multimorbidity.

## Figures and Tables

**Table 1:** Characteristics of participants.

Characteristic		%
Mean age (range), year	58.1 (37–66)	–
Sex	Female	36
	Male	64
Marital status	Married/living with partner	73
	Separated/divorced	27
Education	Grade 8–12	45
	Post-secondary studies or college	45
	University	10
Annual income, CAD	30,000–39,000	45
	50,000+	55
Average number (range) of chronic conditions	7 (5–11)	
Chronic conditions	Artherosclerosis	18
	Arthritis	45
	Asthma	18
	Back pain	18
	Cancer	18
	Chronic obstructive pulmonary disorder	27
	Coronary artery disease	64
	Depression	18
	Diabetes	73
	Hyperlipidemia	91
	Hypertension	91
	Kidney disease	18
	Obesity	55
	Sleep apnea	18
	Other conditions	9

**Table 2:** Themes and sub-themes comprising the fundamental structure of the multimorbidity experience.

Themes	Sub-themes
Impression of premature aging	Chronic diseases symbolizing old age
	Lived experience linked to the loss of physical and social abilities
Unique nature of the experience of multimorbidity	Caught in the sequence of several concomitant diseases
	Multiple interactions: disease–disease; treatment–disease; treatment–treatment
	Diseases experienced differently depending on the presence or absence of pain
Complex management of multimorbidity	Daily burden of multimorbidity
	Difficulties with access to the healthcare system
	Need for support in treatment follow-up
Psychological burden	Negative emotional impacts
	Uncertainty and anticipation related to the fluctuations in health status
Ownership of multimorbidity	Day-to-day adjustment to health problems
	Benefits of self-care

## References

[1] Rogers A, May C, Oliver D (2001). Experiencing depression, experiencing the depressed: the separate worlds of patients and doctors. J Ment Health.

[2] Fortin M, Bravo G, Hudon C, Vanasse A, Lapointe L (2005). Prevalence of multimorbidity among adults seen in family practice. Ann Fam Med.

[3] Schneider KM, O’Donnell BE, Dean D (2009). Prevalence of multiple chronic conditions in the United States’ Medicare population. Health Qual Life Outcomes.

[4] The Commonwealth Fund The Commonwealth Fund 2010 International Health Policy Survey in Eleven Countries.

[5] Barnett K, Mercer SW, Norbury M, Watt G, Wyke S, Guthrie B (2012). Epidemiology of multimorbidity and implications for health care, research, and medical education: a cross-sectional study. Lancet.

[6] Fortin M, Stewart M, Poitras ME, Almirall J, Maddocks H (2012). A systematic review of prevalence studies on multimorbidity: toward a more uniform methodology. Ann Fam Med.

[7] Bayliss EA, Ellis JL, Steiner JF (2007). Barriers to self-management and quality-of-life outcomes in seniors with multimorbidities. Ann Fam Med.

[8] Fortin M, Lapointe L, Hudon C, Ntetu AL, Maltais D, Vanasse A (2004). Multimorbidity and quality of life in primary care: a systematic review. Health Qual Life Outcomes.

[9] Fortin M, Bravo G, Hudon C, Lapointe L, Dubois MF, Almirall J (2006). Psychological distress and multimorbidity in primary care. Ann Fam Med.

[10] Bayliss EA, Steiner JF, Fernald DH, Crane LA, Main DS (2003). Descriptions of barriers to self-care by persons with comorbid chronic diseases. Ann Fam Med.

[11] Morris RL, Sanders C, Kennedy AP, Rogers A (2011). Shifting priorities in multimorbidity: a longitudinal qualitative study of patient’s prioritization of multiple conditions. Chronic Illn.

[12] Leach CR, Schoenberg NE (2008). Striving for control: cognitive, self-care, and faith strategies employed by vulnerable black and white older adults with multiple chronic conditions. J Cross Cult Gerontol.

[13] Roberto KA, Gigliotti CM, Husser EK (2005). Older women’s experiences with multiple health conditions: daily challenges and care practices. Health Care Women Int.

[14] Schoenberg NE, Leach C, Edwards W (2009). “It’s a toss up between my hearing, my heart, and my hip”: prioritizing and accommodating multiple morbidities by vulnerable older adults. J Health Care Poor Underserved.

[15] Schoenberg NE, Bardach SH, Manchikanti KN, Goodenow AC (2011). Appalachian residents’ experiences with and management of multiple morbidity. Qual Health Res.

[16] Sells D, Sledge WH, Wieland M, Walden D, Flanagan E, Miller R (2009). Cascading crises, resilience and social support within the onset and development of multiple chronic conditions. Chronic Illn.

[17] Townsend A, Wyke S, Hunt K (2006). Self-managing and managing self: practical and moral dilemmas in accounts of living with chronic illness. Chronic Illn.

[18] Colaizzi PF, Valle RS, King M (1978). Psychological research as the phenomenologist views it. Existential-phenomenological alternatives for psychology.

[19] Gearing RE (2004). Bracketing in research: a typology. Qual Health Res.

[20] Giorgi A, Giorgi B, Smith JA (2003). Phenomenology. Qualitative psychology: a practical guide to research methods.

[21] Giorgi A, Giorgi A (1985). The phenomenological psychology of learning and the verbal learning tradition. Phenomenology and psychological research.

[22] Penrod J, Gueldner SH, Poon LW, Sprouse BM, Poon LW, Gueldner SH (2003). Managing multiple chronic health conditions in everyday life. Successful aging and adaptation with chronic disease.

[23] Shephard RJ Aging and exercise.

[24] Ward RA (2010). How old am I? Perceived age in middle and later life. Int J Aging Hum Dev.

[25] Ministère de la Famille et des Ainés Preparing the future with our seniors.

[26] Leka S, Griffiths A, Cox T (2004). Work organisation and stress: systematic problem approaches for employers, managers and trade union representatives. Protecting workers’ health series no 3.

[27] Bertalanffy LV (1969). General system theory: foundations, development, applications.

[28] Hudon C, Fortin F, Pélisser-Simard L (2008). Jongler avec la complexité. Le patient atteint de maladies chroniques multiples. Le Médecin du Québec.

[29] Corbin J, Strauss A (1988). Unending work and care.

[30] Boyd CM, Fortin M (2010). Future of multimorbidity research: how should understanding of multimorbidity inform health system design. Public Health Rev.

[31] Registered Nurses Association of Ontario (2002). Establishing therapeutic relationships.

[32] Pollock K, Radley A (1993). Attitude of mind as a means of resisting illness. Worlds of illness: biographical and cultural perspectives on health and disease.

[33] Paterson BL (2001). The shifting perspectives model of chronic illness. J Nurs Scholarsh.

[34] Creswell JW (2007). Quantitative inquiry and research design: choosing among five approaches.

